# Natural Products in Alzheimer’s Disease: A Systematic Review of Clinical Trials and Underlying Molecular Mechanisms

**DOI:** 10.3390/ijms262110631

**Published:** 2025-10-31

**Authors:** Maria T. Bayo Jimenez, Lorenzo Rivas-García, Cristina Sánchez-González, Giuseppe Grosso, Vivian Lipari, Laura Vera-Ramírez, Maurizio Battino, Francesca Giampieri, José L. Quiles, Tamara Y. Forbes-Hernández

**Affiliations:** 1 Department of Physiology, Institute of Nutrition and Food Technology “José Mataix Verdú”, Biomedical Research Centre, University of Granada, Avda. del Conocimiento s.n, 18100 Armilla, Spain; maitebaji@gmail.com (M.T.B.J.); lorenrivas@ugr.es (L.R.-G.); crissg@ugr.es (C.S.-G.); lvera@ugr.es (L.V.-R.); tforbes@ugr.es (T.Y.F.-H.); 2 Department of Biomedical and Biotechnological Sciences, University of Catania, 95123 Catania, Italy; giuseppe.grosso@unict.it; 3 Research Group on Food, Nutritional Biochemistry and Health, Universidad Europea del Atlántico, 39011 Santander, Spain; vivian.lipari@uneatlantico.es (V.L.); m.a.battino@staff.univpm.it (M.B.); f.giampieri@univpm.it (F.G.); 4 Department of Health, Nutrition and Sport, Universidad Internacional Iberoamericana, Campeche 24560, Mexico; 5 Dipartimento di Scienze Cliniche Specialistiche, Facoltà di Medicina, Università Politecnica delle Marche, 60131 Ancona, Italy; 6 Joint Laboratory on Food Science, Nutrition, and Intelligent Processing of Foods, Polytechnic University of Marche, Italy, Universidad Europea del Atlántico Spain and Jiangsu University, China at the Polytechnic University of Marche, 60131 Ancona, Italy; 7 International Joint Research Laboratory of Intelligent Agriculture and Agri-Products Processing, Jiangsu University, Zhenjiang 212013, China; 8 International Research Center for Food Nutrition and Safety, Jiangsu University, Zhenjiang 212013, China

**Keywords:** amyloid, cognitive impairment, dementia, flavonoids, neuroprotection, omega-3 fatty acids, oxidative stress, phenolics, tau

## Abstract

This systematic review included 31 clinical trial articles examining the effects of natural compounds on Alzheimer’s disease (AD) and mild cognitive impairment (MCI), involving 3582 participants aged 50–90. Treatment durations ranged from 8 weeks to 2 years, with an average of 12.5 months. Notably, 11 studies focused on herbal extracts highlighting their prominence in current research. These extracts showed potential cognitive and neuroprotective benefits, although results varied across compounds and study designs. Other natural compounds—including flavonoids, polyphenols, omega-3 fatty acids, *Aloe vera*, *Spirulina*, and citrus phytochemicals—may provide cognitive and neuroprotective benefits, with ginseng and *Ginkgo biloba* combinations also showing promise. *Curcumin* and *Melissa officinalis* had limited effects, resveratrol showed mixed outcomes with some side effects, and matcha green tea may improve cognition and sleep quality. Despite generally favorable results, the studies varied considerably in design and quality; nonetheless, herbal extracts represent a prominent category of natural interventions in AD and MCI, underscoring the need for further large-scale, high-quality clinical trials to confirm their therapeutic potential.

## 1. Introduction

Alzheimer’s disease (AD) is one of the most common forms of dementia and the most widely known degenerative disease [1,2]. It is the fourth leading cause of death after stroke, heart disease, and cancer [3]. AD is characterized by a progressive loss of memory, deterioration of virtually all intellectual functions, increased apathy, decreased speech function, disorientation, and gait irregularities [1]. Moreover, after the age of 65 years old, the risk of developing AD increases, with the risk doubling every 5 years [1,4]. Despite significant research, not much is known about strategies to ameliorate the progression of cognitive decline and the onset of dementia. According to the organization Alzheimer’s Disease International, there were over 55 million people worldwide living with dementia in 2020, especially AD, and it is estimated that in 2030 that number will reach 78 million and will almost double by 2050 [5]. Moreover, some people do not exhibit explicit clinical symptoms of dementia but develop cognitive impairment as the clinical transition phase between normal aging and dementia [6]. This condition is known as mild cognitive impairment (MCI), and it has been associated with an increased risk of AD and considered as a precursor to Alzheimer’s-type dementia, as brains of MCI patients exhibit many pathological features mirroring those of AD subjects [7,8]. Almost 10–15% of MCI cases progress to AD in the range of one year, and 30–50% of cases move to AD after 5–6 years [9,10]. If the MCI is of the amnestic type (amnestic mild cognitive impairment), these numbers go up to 80% [7]. Thus, it is important to prevent and manage AD through early diagnosis at the MCI stage with no clinical symptoms [11]. As mentioned above, the main clinical manifestations of dementia are memory loss and progressive cognitive dysfunction, accompanied by a variety of mental and behavioral abnormalities and personality changes. In 1996, the International Psychogeriatric Association defined the mental and behavioral disturbances known as behavioral and psychological symptoms of dementia (BPSD) as serious and growing public health problems [12]. Successively, in 2004 the Cache County Study on Memory in Aging, which is a longitudinal, population-based study of AD, showed that 67% of dementia subjects with clinically significant symptoms presented at least one or more form of BPSD. Moreover, behavioral aberrancy and neuropsychiatric symptoms such as depression, apathy, psychosis, agitation, and aggression are observed more frequently in moderate to severe AD [13].

There are several hypotheses explaining the pathophysiology of AD, including the cholinergic hypothesis [14], amyloid hypothesis [15], and Tau hypothesis [16]. Hereditary factors have also been considered relevant [17]. From a pathophysiological point of view, the most explored neuropathological hallmark of AD and a potential cause of neuronal damage are the deposition of parenchymal and vascular Amyloid β-peptide (Aβ) in the brain and neurofibrillary tangles formed by the microtubule-associated protein Tau (Tau) [1,18]. It has been demonstrated that 25 years before the first symptoms appear, the deposition of Aβ aggregates in the brain take place; for this reason it has been considered as a major point of interest to propose treatments targeting Aβ aggregation, including formation of neurotoxic Aβ oligomers [19,20]. On the other hand, the tau hypothesis suggests that an excessive or abnormal phosphorylation of tau, which is a highly soluble microtubule-associated protein, results in the transformation of normal tau into PHF-tau (paired helical filament) and neurofibrillary tangles (NFTs). When tau protein loses its solubility structure due to hyperphosphorylation, the new structure damages cytoplasmic functions and interferes with axonal transport, which can lead to cell death and promote the development of dementia [21]. Finally, the hypothesis of cholinergic loss is based on the degeneration of the basal nucleus of Meynert’s cholinergic neurons and of the axons that project to the cerebral cortex. Failure in the cholinergic system impairs its neural function in memory, learning, and other essential aspects of cognition and plays a broader role in promoting neural plasticity [22,23,24].

Unfortunately, there is not yet a well-established therapy for AD. However, certain medicines offer modest benefits, and may be divided into three classes, according to whether they prevent the development of the disease, retard its progression, or offer some symptomatic relief [25]. Currently, the most widely used drugs for the treatment of AD which have shown beneficial effects on standard measures of cognitive function in patients with mild to severe AD are acetylcholinesterase inhibitors such as donepezil, rivastigmine, and galantamine, as well as N-Methyl-D-aspartate blockers, such as memantine [26]. Despite having modest symptomatic effects, these drugs do not have profound effects in the biology of the disease, their efficacy is limited, and they cause numerous adverse reactions, including cognitive impairment, somnolence, unexplained increased mortality rate, extrapyramidal symptoms, and gait disturbance [27,28]. Thus, several studies have been looking for new novel strategies for AD therapy, not only addressing dementia prior to the onset of clinical symptoms but also developing therapeutics for post diagnostic use.

Now, the most viable of these novel therapies is targeting the disruption of neurotransmitter systems. Counteracting the overproduction of Aβ is also an attractive theory and has fostered the development of secretase inhibitors as well as active and passive immunization techniques. Additionally, preclinical studies have demonstrated the neuroprotective potential of various natural compounds in models of AD and neurodegeneration. For example, *Curcumin*, a polyphenol derived from turmeric, has been shown to reduce amyloid plaque accumulation, decrease neuroinflammation, and improve cognitive performance in transgenic mouse models. Similarly, extracts from *Ginkgo biloba* protect neurons against β-amyloid toxicity by enhancing mitochondrial function and reducing oxidative stress, which translates into improved memory outcomes in animal studies [29,30]. Other compounds like resveratrol (RES) activate key cellular pathways such as SIRT1, promoting autophagy and mitochondrial health, thus reducing protein aggregation and oxidative damage [31]. Citrus flavonoids [32], *Curcumin* [33], and sesame seed [34,35] extracts exhibit antioxidant and anti-inflammatory effects, which help preserve synaptic plasticity and neuronal survival.

These findings underscore the multifaceted mechanisms by which natural products may attenuate Alzheimer’s pathology, although clinical validation remains necessary. Collectively, these preclinical investigations provide valuable insights into how natural compounds modulate oxidative stress, neuroinflammation, protein aggregation, and neurotransmitter systems. This evidence supports the therapeutic potential of natural products and phytochemicals in neurodegenerative diseases, emphasizing the importance of further research to translate these findings into clinical applications. Emerging evidence suggests that dietary factors may play a role in brain health and cognitive disorders [36,37,38,39]. Some natural products such as *Ginkgo biloba* extract EGb 761 [7], Nutt grass (*Cyperus rotundus* L.) [40], *Salvia officinalis* and *Melissa officinalis* [41,42], ginger (*Zingiber officinale*) [43], Sweet sedge (*Acorus calamus* L.) [44], black pepper (*Piper nigrum* L.) [45], and incense (*Boswellia serrata*) [46], have shown beneficial effects on the progression of dementia or cognitive impairment in several pre-clinical studies. Dietary antioxidant nutrients, such as vitamins [47] and polyphenols [48], have been shown to potentially be involved in age-related cognitive decline or later stage cognitive impairment, for instance, antioxidant vitamins (vitamins A, C, and E) and homocysteine-related vitamins (vitamins B6, B12, and B9) [49]. Furthermore, polyphenols from olive oil and grapes [50,51], olive fruit extract rich in hydroxytyrosol [52], olive leaf extract enriched in oleuropein [53], strawberry extract [54], Manuka honey and beeswax [55,56], and *Curcumin* [57] have been able to modulate tau hyperphosphorylation and Aβ aggregation in in vivo models of AD. Moreover, a diet rich in fruits and vegetables has been associated with improved cognitive function and a reduced risk of dementia and AD [58]. Plant-derived nutraceuticals present a cost-effective and well-tolerated approach to cognitive enhancement, with higher compliance rates compared to synthetic alternatives [59]. These nutraceuticals impact various brain systems involved in cognitive decline. Although experimental studies have demonstrated their benefits for cognitive function [60,61], controlled clinical trials in older adults have provided limited data and require further ex-ploration [62]. Additionally, three notable reviews discuss the relationship between bioactive compounds and AD, though none are systematic. The first, published in the International Journal of Molecular Sciences [63], explores the impact of bioactive compounds on molecular pathways associated with Alzheimer’s, emphasizing their antioxidant and anti-inflammatory properties. The second review, available in the International Journal for Vitamin and Nutrition Research [64], analyses the role of natural compounds and nutrients in preventing cognitive impairment, with a particular focus on their influence on metabolic mechanisms. The third, featured in Cells [65], examines cellular and molecular mechanisms through which bioactive compounds may exert therapeutic effects in neurodegenerative diseases. Collectively, these reviews provide a foundation for understanding the potential role of nutraceuticals in managing AD, while highlighting the need for more systematic investigations to substantiate their efficacy. However, data from clinical trials are often equivocal and their therapeutic effects are far from satisfactory. Thus, the objective of this systematic review was to analyze the publications of the last 10 years related to clinical trials evaluating the efficacy and safety of natural compounds in AD.

In accordance with all the above-mentioned details, the objective of this systematic review was to critically assess and synthesize the evidence from clinical trials published in the last 10 years that evaluated the efficacy and safety of natural compounds in the treatment or prevention of Alzheimer’s disease. Specifically, the review aimed to accomplish the following: (a) Identify which natural compounds have been investigated in clinical trials for AD and mild cognitive impairment (MCI). (b) Summarize the cognitive and molecular outcomes reported in these trials. (c) Evaluate the methodological quality of the studies and the strength of the evidence supporting therapeutic effects. (d) Highlight existing gaps in clinical research and propose directions for future investigations.

## 2. Materials and Methods

### 2.1. Search Strategy and Selection Criteria

On 21 May 2025, a careful search was performed on the electronic databases PubMed and Web of Sciences to identify clinical trials investigating the relationship between natural compounds and Alzheimer’s disease. Previously, a series of criteria were settled: (i) English-language articles, (ii) clinical trials studies, (iii) research articles evaluating the relationship between Alzheimer’s disease and natural products, and (iv) articles published between 2012 and 2025. A search on the clinicaltrials.gov website (https://clinicaltrials.gov/) was also conducted (accessed on 30 May 2025).

Initially, the key terms used for the article search were “natural products” OR “natural compounds” AND “Alzheimer”. In a second step, the main natural compounds were identified, and a second search was carried out using the terms: “sage” OR “*Curcumin*” OR “olive” OR “*Ginkgo biloba*” OR “berry” OR “rosemary” OR “sesame” OR “chocolate” OR “citrus” OR “aloe” OR “*Spirulina*” OR “herbal extract” OR “grape” OR “caffeine” OR “saffron” OR “ginseng” AND “Alzheimer”.

The initial study selection was based on the examination of titles and abstracts. Studies registered on clinicaltrials.gov were summarized according to the following information: (1) identifier (National Clinical Trial, NCT number), (2) conditions, (3) natural product, (4) intervention design, (5) outcomes, (6) population, (7) locations/country, (8) phase, (9) target/mechanisms, and (10) related publications. The publications indicated in the registered clinical trials were also analyzed. All the records were combined, and duplicates were removed. The full texts of all potentially relevant articles were further reviewed. Studies that did not meet the inclusion criteria listed in Table 1 were finally excluded. The screening and selection of all articles was carried out by two independent reviewers; in cases of disagreement, a third reviewer participated.

### 2.2. Data Collection

The specific information of the selected articles was extracted and tabulated according to the following items: (1) first author, (2) publication year, (3) natural product, (4) objectives of the study, (5) subjects, (6) age range, (7) baseline cognitive test score, (8) treatment, (9) outcomes, (10) safety and tolerability/adverse side effects, (11) main findings, (12) references, and (13) overall study quality. A meta-analysis was not performed due to the heterogeneity of the available studies.

### 2.3. Quality Assessment

The overall quality of the included trials was assessed according to the Revised Cochrane risk-of-bias tool for randomized trials (RoB 2) [66]. This guideline includes detailed criteria to assess potential bias in five domains: (1) risk of bias arising from the randomization process, (2) risk of bias due to deviations from the intended interventions (effect of assignment to intervention), (3) risk of bias due to missing outcome data, (4) risk of bias in measurement of the outcome, (5) risk of bias in selection of the reported result. For each of these biases, the level of risk was classified as low, medium (some concern), or high. The overall risk of bias was established according to the following criteria: (i) low risk of bias when the study presented low risk of bias for all domains; (ii) medium risk of bias when the study raises some concerns in at least one domain, but was not at high risk for any of them, and (iii) high risk of bias when the study presented high risk of bias in at least one domain or medium risk for multiple domains, in a way that compromised confidence in the outcome.

### 2.4. Critical Appraisal Rationale

A critical appraisal of the included studies was conducted to assess the methodological quality and risk of bias, ensuring that the findings of the systematic review are based on reliable and valid evidence. The RoB 2 was used to evaluate each study across five domains: randomization process, deviations from intended interventions, missing outcome data, measurement of outcomes, and selection of reported results [66,67].

Each study was rated as having low risk, some concerns, or high risk of bias per domain, and an overall risk rating was assigned accordingly. This appraisal informed the interpretation of findings and was considered in the narrative synthesis. Studies with high risk of bias were not excluded but were clearly noted, and their limitations were discussed when drawing conclusions.

### 2.5. Systematic Review Registration

This review has been registered with the International Prospective Register of Systematic Reviews (PROSPERO) under the registration number CRD1112523. The title of the registered review is “Natural Products in Alzheimer’s Disease: A Systematic Review of Clinical Trials and Underlying Molecular Mechanisms”. The registration was completed on 25 July 2025 and last edited on the same date.

### 2.6. PRISMA Statement

This systematic review was conducted and reported in accordance with the PRISMA 2020 (Preferred Reporting Items for Systematic Reviews and Meta-Analyses) guidelines. A completed PRISMA checklist is available in the Supplementary Materials [68]. The PRISMA flow diagram was used to document the study selection process.

### 2.7. Synthesis of Results

The data extracted from the included clinical trials were synthesized narratively due to the heterogeneity in study designs, interventions, durations, dosages, and outcome measures. Key findings were organized thematically according to the natural compounds studied and their reported effects on cognitive outcomes in Alzheimer’s disease (AD) and mild cognitive impairment (MCI).

Where possible, results were grouped by compound type (e.g., polyphenols, flavonoids, and herbal extracts) and mechanism of action (e.g., anti-inflammatory, antioxidant, and anti-amyloidogenic). A descriptive approach was used to compare outcomes, highlight patterns, and identify gaps in the evidence. Risk of bias assessments were incorporated into the interpretation of results to provide context for the strength of the findings.

## 3. Results and Discussion

### 3.1. Study Selection

**Figure 1:**
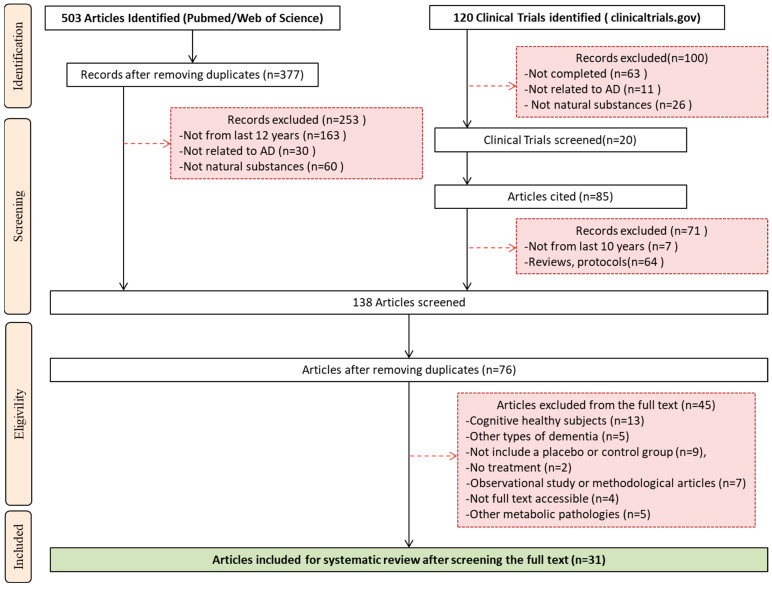
Flowchart for the screening and selection of articles. **Black arrows** indicate the studies that were carried forward, while **dashed red arrows** indicate the studies that were excluded.

**Figure 2:**
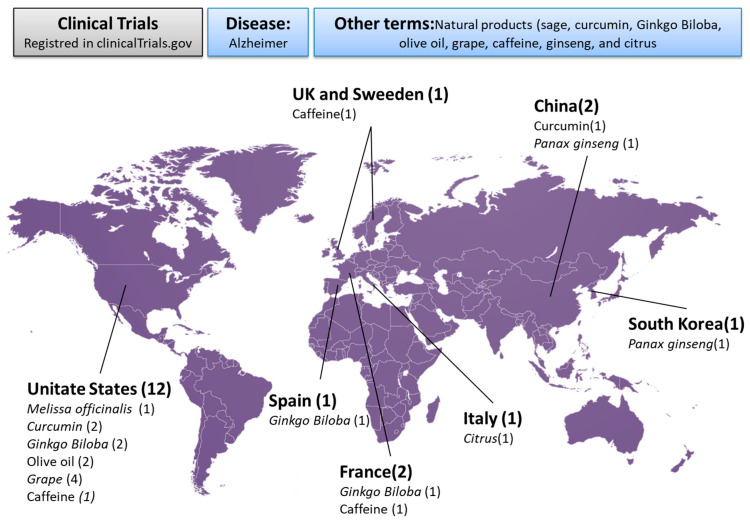
Location of completed clinical trials registered on ClinicalTrials.gov.

A systematic search in the electronic databases PubMed and Web of Science identified 503 articles. After removing duplicates (*n* = 126) and screening titles and abstracts, 253 additional articles were excluded based on the following criteria: (i) published before 2014 (*n* = 163), (ii) not related to AD (*n* = 30), and (iii) not involving natural compounds (*n* = 60). Similarly, among the 120 clinical trials registered in the clinicaltrials.gov website, 99 studies were excluded for the following reasons: (i) not completed (*n* = 63), (ii) not related to AD (*n* = 11), or (iii) non-natural compounds were tested (*n* = 26) (Figure 1). The information on the remaining 20 studies is summarized in Table 2. Additionally, Figure 2 illustrates the geographic distribution of completed clinical trials registered on ClinicalTrials.gov, highlighting that most trials were conducted in the United States, where studies involving grape-derived compounds or RES were predominant as well as studies that evaluated the effectiveness of *Ginkgo biloba* in the treatment of AD. The information about them on the website is sparse and most of the articles related were excluded as discussed below. From these 20 studies, 85 articles were further identified and were also reviewed. Among them, 71 articles were excluded due to the following reasons: (i) published prior to the year 2012 (*n* = 7) or (ii) review articles or methodological papers (*n* = 64). The total number of articles screened at this point from PubMed, Web of Sciences, and the clinicaltrials.gov website was 138 articles. After eliminating duplicates and reviewing titles and abstracts, 62 other articles were excluded.

Finally, a thorough full-text review was conducted on 76 articles, of which 45 were excluded for the following reasons: (i) treatment was evaluated in cognitively healthy subjects (*n* = 13), (ii) treatment was assessed in patients with other types of dementia (*n* = 5), (iii) the experimental design did not include a placebo or control group (*n* = 9), (iv) no treatment was evaluated, only AD biomarkers were studied (*n* = 2), (v) observational or methodological studies (*n* = 7), (vi) full text was not accessible (*n* = 4), and (vii) treatment was assessed in subjects with other metabolic conditions (*n* = 5). Ultimately, a total of 24 articles were included in the systematic review (Figure 1).

### 3.2. Analysis of Included Studies

**Figure 3:**
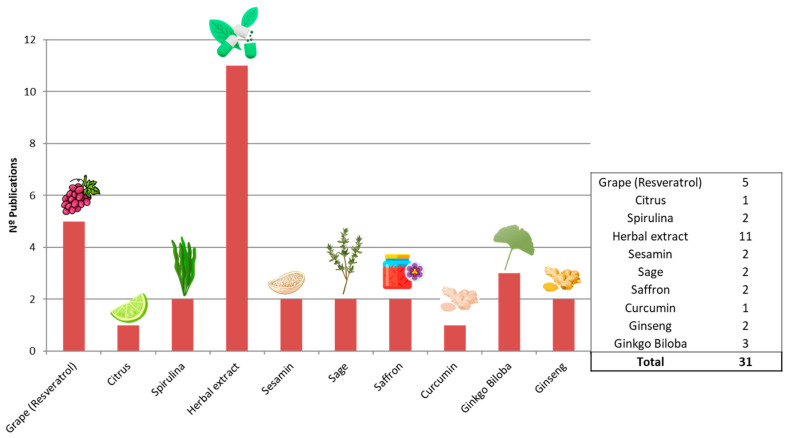
Clinical trial-related articles per natural compound.

Thirty-one clinical trial-related articles investigating the association between natural compounds and AD or MCI were systematically reviewed. Most of them evaluated the safety and effectiveness of herbal extract treatments (*n* = 11), followed by RES + grapes (*n* = 5), saffron (*n* = 2), sesame-related compounds (*n* = 2), *Spirulina* (*n* = 2), *Citrus* (*n* = 1), ginseng (2), *Ginkgo biloba* (3), *Curcumin* (1), and *Melissa ofcinalis (sage)* (*n* = 2) (Figure 3).

**Figure 4:**
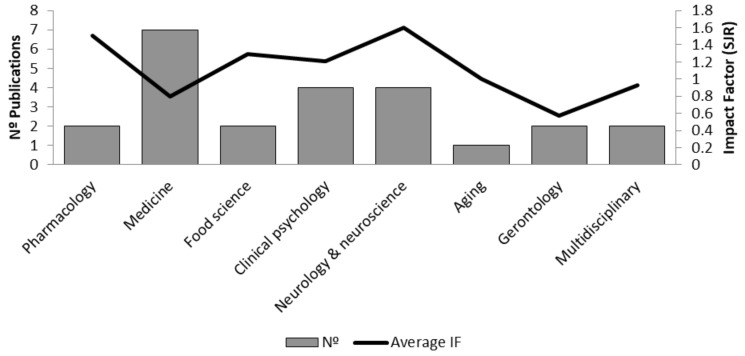
Bibliometric indicators of the selected articles.

Most of the articles were published in journals related to the following categories: medicine, neurology and neuroscience, and clinical psychology. The average impact factor of the publications was 1083. However, 79.16% of them were published in journals within the first quartile (Q1) (Figure 4).

To evaluate the outcomes, different cognitive tests were used, including the Modified Mini-Mental State Examination (MMSE) score [46,47,48,49,50,51,52,53,54,55,56,57,58,59,60,61,62], Neuropsychiatric Inventory (NPI) [51,53,54,56,60], Digit Symbol Substitution test (DSS; subtest of the Wechsler Adult Intelligence Scale) [48,55], immediate and delayed word recall [48], attention span, Functional Activities Questionnaire (FAQ) [48], Geriatric Depression Scale (GDS) [48,60], Montreal Cognitive Assessment (MoCA) [57,59,60,62], ADAS-Cognitive Chinese Medicine Symptom Scale (CM-SS) [57,59], Activities of Daily Living score (ADL) [47,49,50,56,59], Cognitive Subscale of the Alzheimer’s Disease Assessment Scale (ADAS-Cog) [46,49,51,52,53,54,56,57,59,60,61,62], Clinical Dementia Rating Scale Sum of Boxes (CDR-SOB) [53,60,61], Auditory Verbal Learning Test (AVLT) [52,58], Rey-Osterrieth Complex Figure test (ROCF) [55,58], Seoul Neuropsychological Screening Battery (SNSB) [61], Computerized Neurocognitive Function Test (CNT) [61,62], Benton Visual Retention Test [55], Functional Independence Measure (FIM) [62], Severe Cognitive Impairment Rating Scale (SCIRS) and Functional Assessment Staging (FAST) [69], and Central Nervous System Vital Signs (CNSVS) [56]. Neuropsychological tests are very useful for the diagnosis, evaluation, and prognosis of Alzheimer’s disease (AD). Among them all, the most widely used are the MMSE and ADAS-Cog scores. The MMSE evaluates different mental abilities, including orientation, short-term memory, attention and concentration, and visuospatial and language skills, as well as the ability to understand and follow instructions [50,62]. On the other hand, the ADAS-Cog is a more comprehensive tool, consisting of around 11 items, including word recall and recognition, object naming, comprehension, following and remembering instructions, spoken language, ideational apraxia, and tasks evaluating orientation and attention [62]. The scores obtained from these tests are closely linked to AD severity [50]. An MMSE score ≥ 25 is considered normal, while scores below 24 indicate possible cognitive impairment [62]. Conversely, higher scores on the ADAS-Cog (ranging from 0 to 75 points) reflect a greater degree of impairment [59,62].

We included a newly introduced cognitive tests summary table (Table 3) designed to provide a succinct and organized overview of the cognitive assessments discussed.

The main characteristics of the studies are discussed in the following sections according to the natural compound (Table 4).

### 3.3. Herbal Extracts

Eleven of the thirty-one articles included in the review evaluated the effects of traditional herbal supplementation on Alzheimer’s disease (AD) or mild cognitive impairment (MCI), with none of the formulations being identical (Table 4). Despite the considerable variation in composition, several compounds were used in at least two studies: Japanese angelica root [56,60], Glycyrrhiza [56,60], *Ligusticum wallichii* [52,59], *Panax ginseng* [56,59], and Radix *Polygoni multiflori* [50,52,57]. These studies included a total of 799 patients, with an average of 36 individuals per experimental group. Participant ages ranged from 50 to 85 years. The average treatment duration was 12.5 months, ranging from 3 months to 6 years. Five of the studies used a placebo group as control [54,58,60,61,63], while the remaining six compared the herbal treatment to donepezil [52,56,57,59], administered at either 5 mg/day or 20 mg/day [48], or to piracetam (2.4 g/day) [50]. Most studies (91%) reported that herbal supplementation improved cognitive function in patients with AD or MCI [48,50,52,53,54,56,57,58,59,61,63]. Only one study (~9%) showed no significant cognitive improvement [60]. Several interventions demonstrated clear benefits over placebo. For example, Davaie Loban (DL) capsules improved general cognition and memory, reducing ADAS-Cog and CDR-SOB scores after three months of treatment (500 mg, three times daily) [54]. Bushen capsules (BSC) improved MMSE, ROCF recall, and AVLT scores after 3–12 months of supplementation [58,59] and were also associated with normalized activation of the left superior parietal lobe during working memory tasks [59]. Matcha green tea (natural) was evaluated to assess its effect on cognitive function and sleep quality in older adults with cognitive decline, showing positive effects on both outcomes [70]. Kami-guibi-tang (KGT) significantly improved CDR-SB and SNSB-D scores in MCI patients [71]. In contrast, the Yokukansan (YKS, TJ-54) herbal formula showed no significant effects on psychological symptoms or MMSE scores compared to placebo [60]. Three studies also showed that herbal formulations outperformed donepezil. For example, a polyherbal formulation *containing Bacopa monnieri, Hippophae rhamnoides, and Dioscorea bulbifera* (1 g/day for one year) improved DSS, FAQ, and GDS scores, although MMSE scores remained unchanged [48]. The Yishen Huazhuo decoction (YHD) (62 g/day for 24 weeks) [52] and Ninjin’yoeito (NYT) (7.5 g/day for 24 months) [56] showed significantly better outcomes than donepezil (5 mg/day) in ADAS-Cog and MMSE scores. Other formulations such as the Jiannao Yizhi Formula (JYF) [59] and Huannao Yicong Formula (HYF) [57] showed similar efficacy to donepezil in improving cognition, memory, and language. All treatments increased MMSE and MoCA scores and decreased CM-SS and ADAS-Cog scores from baseline. Bushenhuatanyizhi (BHY) instant granules (12 g/day) improved MMSE and ADL scores comparably to piracetam [50]. Only four studies evaluated biochemical biomarkers related to AD. One reported that the polyherbal formulation markedly reduced oxidative stress and inflammation, evidenced by lower TBARS, GPx, CRP, homocysteine, and TNF-α, along with increased GSH levels [48]. Similarly, BHY granules reduced lipid peroxidation [50], while JYF [59] and HYF [57] reduced serum levels of tau, Aβ42, and AchE. These effects may underline the observed cognitive improvements. A more recent study by Uchida et al. (2024) investigated the effect of matcha green tea on cognition and sleep in older adults with early cognitive decline. Over a 12-month randomized controlled trial, matcha (rich in antioxidant catechins) was associated with improved cognitive performance and sleep quality, with no major adverse effects reported [70]. Most of the reviewed studies demonstrated that traditional herbal supplements could enhance cognitive function in AD and MCI, often performing as well as, or better than, established treatments like donepezil. These improvements appear linked to mechanisms involving oxidative stress reduction, anti-inflammatory effects, and lowered amyloid-β levels. However, the heterogeneity of formulations and methodologies across studies underscores the need for standardized, large-scale trials to confirm these findings and clarify the active mechanisms involved.

### 3.4. Resveratrol

RES is the main phenolic compound found in grapes, Cassia plants, mulberries, and blueberries. It exhibits significant antioxidants, anti-inflammatory, cardioprotective, immunomodulatory, and anticancer properties [55,72,73,74,75,76]. Growing evidence has highlighted the potential role of RES in the prevention and treatment of neurodegenerative disorders, including AD [77]. Four of the articles included in this systematic review evaluated the safety and efficacy of RES in patients with Alzheimer’s disease (AD), and one assessed the effects of a grape formulation on cerebral metabolism in patients with mild cognitive impairment (MCI). In total, these five studies involved 380 subjects with a minimum age of 49 years. The intervention periods ranged from 8 to 52 weeks. Three studies suggested that RES may slow progressive cognitive decline in AD and MCI subjects [62,78,79], whereas one reported no significant changes [80], and another observed negative outcomes [53]. In a 52-week trial, high doses of RES (500–2000 mg/day) markedly increased plasma matrix metalloproteinase (MMP)-10 levels and cerebrospinal fluid (CSF) levels of interleukin (IL)-4, macrophage-derived chemokine (MDC), and fibroblast growth factor (FGF)-2 compared to placebo. Additionally, RES reduced CSF MMP-9 and amyloid-beta 42 (Aβ42) levels. These results suggest modulation of neuroinflammation and induction of adaptive immunity in AD patients. The reduction in MMP-9 may indicate that RES helps maintain central nervous system (CNS) permeability, potentially limiting infiltration of inflammatory agents such as leukocytes into the brain. Cognitively, RES treatment attenuated declines in MMSE scores and changes in activities of daily living (ADL) and ADAS-Cog scores [78]. Conversely, another 52-week study with similar doses found that plasma and CNS Aβ40 levels were lower in the placebo group than in the RES-treated group, indicating no beneficial effects. While RES slowed brain volume loss, this was not accompanied by cognitive or functional improvement, and no effects on plasma or CNS Aβ42 and tau levels or ADAS-Cog, MMSE, Clinical Dementia Rating (CDR), and Neuropsychiatric Inventory (NPI) scores were observed compared to the placebo [53]. Further, combining RES (1000–2000 mg/day) with donepezil (5 mg/day) significantly improved MMSE, Functional Independence Measure (FIM), and ADAS-Cog test results in AD patients compared to donepezil alone. It also reduced inflammatory biomarkers, including IL-6, tumor necrosis factor-alpha (TNF-α), and Alzheimer-associated neuronal thread protein (AD7C-NTP) levels [62]. In contrast, a study administering a daily oral preparation containing RES (10 mg), glucose (5 g), and malate (5 g) for 12 months found no significant changes in MMSE, ADAS-Cog, or NPI scores compared to a non-treated group [81]. Finally, daily consumption of 72 g of grapes for 6 months demonstrated positive effects on brain metabolism in MCI patients. Although no significant differences were noted in ADAS-Cog, MMSE, and Rey-Osterrieth Complex Figure (ROCF) scores between the grape and placebo groups, significant regional cerebral metabolic declines in the cingulate cortex, left superior posterolateral temporal, and left prefrontal areas were observed only in the placebo group. These effects may be attributed to the high RES content of grapes [79].

Taken together, these findings are contradictory and inconclusive. Several mechanisms have been proposed to explain RES’s therapeutic effects against AD. These include inhibition of inflammatory responses [79,81,82], improvement of antioxidant status [62,79], inhibition of Aβ formation [62], promotion of brain resilience to Aβ deposition [78], maintenance of Blood–brain barrier (BBB) integrity through MMP-9 reduction [78], sirtuin 1 activation [53,78], autophagy promotion [53], and a coordinated peripheral and central immune response that may inhibit neuronal death [78]. Of the five included trials, three reported cognitive benefits, and two reported negative outcomes. The most significant adverse effects included weight loss [78], nausea, and diarrhea [53], but these were like those observed in placebo groups. The variability in results highlights the need for further research to establish clear conclusions. Future studies should address inconsistencies, explore optimal dosages and treatment durations, and clarify mechanisms of action to determine the true potential of RES as a therapeutic intervention for cognitive decline.

### 3.5. Saffron

Recent evidence suggests that saffron may hold potential as a therapeutic option for cognitive disorders, prompting several clinical investigations into its efficacy [83]. One of the two included trials related to saffron evaluated its efficacy in the treatment of mild to moderate AD [69], while the other assessed its efficacy in managing cognitive impairment in patients with multidomain MCI [80]. In the first study, supplementation with 30 mg/day saffron extract for 12 months showed similar efficacy to 20 mg/day memantine in reducing cognitive decline, as demonstrated by changes in Severe Cognitive Impairment Rating Scale (SCIRS) and Functional Assessment Staging (FAST) scores. Saffron capsules also exhibited a favorable safety profile [69]. The second study reported that saffron treatment for 1 year improved MMSE scores compared to a placebo, although the dose was not specified. Both studies involved fewer than 35 saffron-treated participants, which limits generalizability [80]. Saffron, the dried stigma of Crocus sativus L. (Iridaceae), contains crocin, crocetin, picrocrocin, and safranal as main secondary metabolites [69]. Although mechanisms were not directly studied, neuroprotective effects were suggested to result from inhibition of β-amyloid peptide aggregation and acetylcholinesterase (AChE) activity, increasing acetylcholine levels [69]. Preclinical studies further support saffron’s role in enhancing synaptic plasticity and mitigating ethanol-induced hippocampal dysfunction, which may underlie cognitive benefits [80,84]. A limitation in both studies was the small sample size (fewer than 35 saffron-treated participants), which reduces the generalizability of findings. While the studies did not directly investigate mechanisms of action, previous research suggests saffron’s neuroprotective effects may involve inhibition of β-amyloid aggregation and AChE activity, increasing acetylcholine levels. Previous pre-clinical studies have shown that the administration of a crocin extract counteracted ethanol inhibition of N-methyl-D-aspartate receptor-mediated responses in rat hippocampal neurons and prevented ethanol-induced inhibition of hippocampal long-term potentiation, a form of activity-dependent synaptic plasticity that may trigger learning and memory, which further supports saffron’s role in enhancing synaptic plasticity and mitigating ethanol-induced hippocampal dysfunction, which may underlie its cognitive benefits [1,60]

### 3.6. Sesame-Related Compounds

Sesame (*Sesamum indicum* L., *Pedaliaceae*) is a widely distributed crop in Asia, rich in nutrients and phytochemicals such as sesamin, sesamol, sesamolin, and sesaminol [45,46,85]. Two clinical trials examined sesame compounds’ effects on cognitive function.

Jung et al. [69] studied sesame oil cake extract (SOCE) supplementation in MCI subjects over 12 weeks. SOCE improved verbal learning test scores and decreased plasma Aβ (1–40) and Aβ (1–42) levels compared to placebo. The sesaminol content was 3.1 mg/g, noted as a strong antioxidant [85,86]. Ito et al. [87] reported that supplementation with 10 mg/day sesamin combined with 6 mg/day astaxanthin for 12 weeks improved cognitive functions related to processing speed and complex task performance in MCI patients. The study could not isolate the effect of each compound, but both are recognized free radical scavengers. No significant changes were observed in malondialdehyde, paraoxonase 1 (PON1), or oxidized LDL blood levels. Some patients reported dizziness, colds, or diarrhea [87]. Both studies indicate potential cognitive benefits of antioxidant supplementation in MCI.

### 3.7. Ginseng and Ginkgo biloba

Ginseng, mainly composed of ginsenosides, is a widely used medicinal herb, especially in Asia, with antioxidant and anti-inflammatory properties [46,85]. *Ginkgo biloba* extract contains flavonoids and terpenoids that improve cognition, reduce amyloid deposition, inhibit neurofibrillary tangles, and enhance brain circulation [2,3]. This review included two clinical trials on ginseng and two on *Ginkgo biloba*. One trial investigated *Panax ginseng* effects on cognitive performance in AD patients, measuring MMSE and other scales, along with hematopoietic progenitor cell counts. While minor cognitive improvements were noted, results were inconclusive, indicating the need for further studies [85]. Another trial examined GRAPE granules, a Chinese herbal formulation containing *Panax ginseng* combined with donepezil and memantine. Cognitive assessments (MMSE, ADCS-ADL, and CDR) and biomarker studies suggested some cognitive benefits, but findings were not robust and require confirmation in larger trials [46]. Heo et al. [45] evaluated different doses of the ginseng formula SG-135 in mild to severe AD patients. The 4.5 mg/day dose improved MMSE and ADAS-Cog scores at 12 and 24 weeks. Another study assessed Memo^®^, containing 150 mg ginseng, 120 g *Ginkgo biloba*, and 750 mg lyophilized royal jelly in MCI patients. After 4 weeks, Memo^®^ improved MMSE scores versus placebo, suggesting potential in early cognitive decline [86]. Herrschaft et al. [86] and Ihl et al. [87] showed that *Ginkgo biloba* extract (EGb 761^®^) at 240 mg/day improved cognitive and neuropsychiatric symptoms in mild to moderate AD and vascular dementia. However, a large five-year trial by Vellas et al. [88] found no preventive effect of *Ginkgo biloba* on AD onset in at-risk older adults.

Collectively, ginseng—especially combined with *Ginkgo biloba*—may benefit cognitive function via antioxidant, anti-inflammatory, and neuroprotective mechanisms, though further research with modern methods and larger samples is necessary. All studies reported good tolerability with no serious adverse effects [45,46,85,86,87,88].

### 3.8. Curcumin

*Curcumin* is a phenolic compound derived from the rhizome of the Curcuma longa plant with several biological effects, including antioxidant, anti-inflammatory, and anticancer properties [89,90]. It has also been demonstrated that it is able to reduce Aβ aggregation and modulate tau processing [89]. In fact, *Curcumin* treatment has been effective in improving cognitive and behavioral functions in some animal studies. Ringman et al. demonstrated that *Curcumin* C3 Complex^®^ supplementation (2 or 4 g/day) for 24 weeks had negative outcomes compared to placebo in patients with mild to moderate AD. The supplement was well tolerated, and only minor gastrointestinal problems occurred in some patients (21%) of the treated group. However, its consumption was associated with an increase in blood glucose levels and a decrease in hematocrit. No effects on ADAS-Cog, NPI, or ADL scores or on CSF levels of Aβ (1–42), tau, and p-tau 181 were observed [91].

### 3.9. Melissa officinalis

*Melissa officinalis* is a medicinal plant used worldwide for its therapeutic effects. Its extracts and essential oils are rich in bioactive compounds, including ursolic and oleanolic acids (triterpenes), geranial, geraniol, citronellal and neral (volatile compounds), quercetin, luteolin and rhamnocitrin (flavonoids), and rosmarinic, caffeic, and chlorogenic acids (phenolic acids) [92].

In one of the reviewed studies, Noguchi-Shinohara et al. [60] examined the effects of an extract of *Melissa officinalis* containing 500 mg of rosmarinic acid on cognitive function of patients with mild dementia due to AD, totaling 23 participants. No significant differences were observed between the treated patients and placebo group in terms of MMSE, ADAS-cog, DAD, or CDR scores after 24 weeks. Only the mean NPI-Q score significantly improved after the extract supplementation, suggesting positive effects of the *Melissa officinalis* extract in the management of lability or irritability in AD patients.

The same authors stated that rosmarinic acid is capable of inhibiting Aβ fibrils formation and oligomerization and preventing memory loss in murine models [60]. However, in the clinical trial, they did not assess any biological biomarkers. Most recently in a clinical trial which included a much larger sample of older adults without dementia but with subjective or mild cognitive impairment totaling 323 participants, Noguchi-Shinohara et al. found a significant difference in the Clinical Dementia Rating Sum of Boxes score among participants without hypertension, with the M. officinalis group showing a smaller increase compared to the placebo group, hinting at a potential effect in this subgroup. Additionally, they identified a significant impact on participants without hypertension, suggesting M. officinalis might be beneficial in preventing cognitive decline in this specific population [93].

In summary, a total of 346 patients were included in these studies, with a mean number per experimental group of 36 individuals. The minimum age of the subjects was over 59 years old and the minimum duration of treatment was 24 months [60], with a maximum of 96 months, followed by an additional 24-week washout period, resulting in a longer study duration [93]. Both studies used a randomized, placebo-controlled, double-blind trial design. This design ensures that neither participants nor researchers knew who received the treatment or placebo, which minimizes bias. As well as both examining the effects of M. officinalis extract, which specifically contained 500 mg of rosmarinic acid, administered daily, each study evaluated the safety and tolerability of M. officinalis extract. Both found no serious adverse events or significant changes in vital signs or physical and neurological measures. In contrast, neither study found significant differences in overall cognitive measures between the M. officinalis and placebo groups, indicating that rosmarinic acid did not significantly improve cognitive function in either population over the study period. In summary, both studies investigated M. officinalis extract containing rosmarinic acid and found it safe and well-tolerated [60,93].

### 3.10. Spirulina

*Spirulina maxima* is a microscopic, filamentous cyanobacterium widely used as a nutraceutical food supplement due to its biological properties. It contains many bioactive compounds, including phenols, phycocyanins, and polysaccharides for which antioxidant, anti-inflammatory, and immunomodulatory activities have been demonstrated [94,95]. In recent years, some studies have shown its neuroprotective effects on the development of the neural system and some neurological and neurodegenerative diseases [94,95]. In a clinical trial involving 80 patients with MCI, Choi et al. [96] demonstrated that *Spirulina maxima* 70% ethanol extract (SM70EE) supplementation for 12 weeks significantly improved visual working memory, visual memory, and verbal memory, according to CNT scores, compared to a placebo group. SM70EE treatment also enhanced the score of items related to vocabulary in the MoCA test and the total antioxidant capacity of plasma. Cognitive improvements observed in this study were attributed to the antioxidant effects of SM70EE, which may reduce oxidative stress, a known factor linked to cognitive decline. However, no significant differences were observed between groups in terms of Aβ (amyloid-beta) levels, suggesting that while oxidative stress reduction might explain some of the cognitive improvements, other mechanisms, such as anti-inflammatory or neuroprotective effects, could also be at play. The cognitive improvements observed in the study could be partially attributed to the antioxidant effects of SM70EE, which may reduce oxidative stress, a factor linked to cognitive decline. However, since Aβ levels did not change, the positive cognitive outcomes might involve other mechanisms, such as anti-inflammatory or neuroprotective effects, which were not directly studied. This suggests that while the antioxidant properties of *Spirulina maxima* play a role, additional pathways might contribute to its cognitive benefits. In 2023, Tamtaji et al. investigated the impact of *Spirulina* supplementation on cognitive function and metabolic status in patients with AD. In a 12-week, randomized, double-blind, controlled trial, 60 AD patients were assigned to receive either 500 mg/day *Spirulina* or a placebo (30 participants per group). Cognitive function was assessed using the MMSE, and metabolic markers were measured at baseline and post-intervention. Results showed that *Spirulina* significantly improved MMSE scores compared to placebo (+0.30 vs. −0.38, *p* = 0.01), indicating enhanced cognitive function. Additionally, *Spirulina* supplementation led to improvements in metabolic parameters, though the study did not find significant changes in key neurobiological markers, such as Aβ levels. The improvements in cognitive scores and metabolic health suggest that *Spirulina* might contribute to enhancing cognitive function, potentially through mechanisms not fully explored in the trial, such as anti-inflammatory, neuroprotective, or metabolic pathways [97]. Both studies reported significant cognitive improvements in the *Spirulina* group compared to the placebo group, suggesting that *Spirulina* or its extract may offer benefits in managing dementia or AD symptoms. However, a closer examination of the evaluated biomarkers reveals a more nuanced picture. While cognitive outcomes were positive, some key biomarkers—such as Aβ levels—did not show significant changes, raising questions about the underlying mechanisms. This discrepancy suggests that the cognitive improvements observed might be mediated by alternative pathways not fully explored in these studies. Inflammatory markers, neuroprotective factors, or oxidative stress-related pathways—none of which were comprehensively assessed—could play a critical role. Therefore, further studies are needed to expand the biomarker panel and clarify whether the cognitive benefits of *Spirulina* are explained by currently known mechanisms or if they involve other, as yet unstudied, biological effects.

### 3.11. Citrus

Auraptene (AUR) and naringenin (NAR) are citrus-derived phytochemicals that influence several biological mechanisms associated with cognitive decline, including neuronal damage, oxidative stress, and inflammation [98]. A recent 36-week placebo-controlled clinical trial by Galluzzi et al. aimed to evaluate the cognitive and biological effects of a citrus peel extract rich in AUR and NAR on older adults with SCD. This innovative study combined cognitive assessments with biomarker analysis to measure efficacy and identify mechanisms of action [99]. Cognitive assessments included the Repeatable Battery for the Assessment of Neuropsychological Status (R-BANS) and other cognitive tests, showing improvements in memory and attention. Biological markers such as interleukin-8 (IL-8), brain-derived neurotrophic factor (BDNF), and neurofilament light chain were also analyzed. Results indicated reductions in IL-8 levels and positive cognitive outcomes, likely due to the antioxidant and anti-inflammatory properties of the extract. However, other unexplored mechanisms may have contributed to the observed benefits. The study yielded positive outcomes and provided valuable insights for the development of larger, long-term clinical trials to evaluate the effectiveness of citrus phytochemical supplements in preventing AD. It addressed gaps in clinical evidence for nutraceuticals targeting SCD. However, the focus on individuals with SCD introduced variability in the study population, as SCD does not always correlate with underlying AD pathology. Despite this limitation, the findings provided valuable insights into cognitive decline and established a basis for designing larger, long-term clinical trials. These results also contributed to public health efforts by offering a framework for interventions aimed at older adults at risk of cognitive decline.

**Table 4:** Characteristics of clinical trial-related publications on natural compounds and AD.

First Author and Year	Treatment	Objectives	Subjects	Age	Inclusion Criteria (Cognitive Test Score)	Outcomes Assessment	Adverse Side Effects	Main Findings
Herbal extracts								
[67] Kudoh et al., 2016.	Ninjin’yoeito (NYT) formula (7.5 g/day).	Evaluate efficacy for AD treatment.	30 AD patients.	50–85 years.	MMSE, ADAS-cog, CDR.	MMSE, ADAS-Cog, CDR-SB, MoCA.	Mild gastrointestinal discomfort.	NYT improved ADAS-cog and MMSE scores significantly compared to donepezil.
[58] Wang et al., 2020.	Jiannao Yizhi Formula (JYF) (10 g/day).	Cognitive improvement in AD.	40 AD patients.	50–85 years.	ADAS-Cog, MMSE, MoCA.	ADAS-Cog, MMSE, MoCA.	Mild headache, dizziness.	JYF demonstrated similar efficacy to donepezil in improving cognitive function.
[50] Zhang et al., 2015.	Bushen capsules (BSC) (varied dose).	MCI cognitive improvement.	30 MCI patients.	50–85 years.	MMSE, AVLT, ROCF.	MMSE, AVLT, ROCF.	No adverse side effects noted.	BSC significantly improved memory and cognitive function after 3–12 months.
[53] Furukawa et al., 2017.	Yokukansan (YKS, TJ-54).	Evaluate efficacy in psychological symptoms of AD.	35 AD patients.	50–85 years.	NPI-Q, MMSE.	NPI-Q, MMSE.	No adverse effects.	No significant improvement in psychological symptoms of dementia.
[47] Sadhu et al., 2014.	Polyherbal formula (1 g/day).	Evaluate cognitive improvement in AD.	50 AD patients.	50–85 years.	DSS, FAQ, GDS.	DSS, FAQ, GDS.	No significant adverse effects.	Polyherbal formulation improved cognitive function better than donepezil.
[51] Zhang et al., 2015.	Yishen Huazhuo decoction (62 g/day).	Cognitive function in AD.	60 AD patients.	50–85 years.	ADAS-cog, MMSE.	ADAS-cog, MMSE.	No significant adverse effects.	YHD outperformed donepezil in improving cognitive scores.
[56] Yang et al., 2019.	Huannao Yicong Formula (HYF) (10 g/day).	Cognitive improvement in AD.	40 AD patients.	50–85 years.	MMSE, MoCA, CM-SS.	MMSE, MoCA, CM-SS.	No significant adverse effects.	HYF had similar efficacy to donepezil in improving cognitive scores.
[71] Shin et al., 2021.	Kami-guibi-tang (KGT).	Improve cognition and memory in AD.	45 AD patients.	50–85 years.	CDR-SB, SNSB-D.	CDR-SB, SNSB-D.	No adverse effects.	KGT significantly improved CDR-SB and SNSB-D scores.
[62] Zhang et al., 2019.	Bushen capsules (BSC).	Cognitive function in aMCI.	35 MCI patients.	50–85 years.	MMSE, AVLT, ROCF.	MMSE, AVLT, ROCF.	No significant adverse effects.	BSC significantly improved cognitive functions in aMCI patients after 12 months.
[70] Uchida, K. et al., 2024	Matcha green tea (natural)	To assess the effect of matcha green tea on cognitive function and sleep quality in older adults with cognitive decline.	Older adults with cognitive decline.	60–85 years	Cognitive decline (MMSE < 26).	Cognitive function tests, sleep quality assessment.	No major adverse effects reported.	Matcha green tea showed positive effects on cognitive functions and sleep quality.
Resveratrol								
[74] Moussa et al., 2017.	RES (500–2000 mg/day) for 52 weeks.	To evaluate the safety and efficacy of RES in AD patients.	100 AD patients.	≥49 years.	MMSE ≤ 24, ADAS-Cog ≥ 14.	MMSE, ADAS-Cog, CSF markers, plasma MMP-9, Aβ42 levels.	Weight loss (like placebo group).	RES slowed cognitive decline, improved MMSE, and reduced CSF MMP-9 and Aβ42 levels. Suggests neuroinflammation modulation and immune activation in AD patients.
[75] Li B. et al., 2023.	Grape seed procyanidins extract (GSPE).	To evaluate the effect of GSPE on cognitive function in elderly individuals with mild cognitive impairment (MCI).	71 participants (35 GSPE, 36 placebo).	≥60 years.	Diagnosis of MCI.	Montreal Cognitive Assessment (MoCA).	No significant adverse effects reported.	No significant improvement in cognitive function with GSPE supplementation over 6 months compared to placebo.
[61] Liu X et al., 2025	Resveratrol (vs. placebo)	To determine whether resveratrol modulates CSF biomarkers of neurodegeneration, inflammation, microglial activation in Alzheimer’s disease.	Placebo (*n* = 21) vs. resveratrol (*n* = 30) from a prior multicenter trial	older adult age range	Participants from prior multicenter phase 2 trial of AD — mild-to-moderate Alzheimer’s disease	Biomarkers in CSF: neuron-specific enolase (NSE), phosphorylated neurofilaments (PNF), cathepsin D, MMP-9, TREM2, angiogenin, others.	None significantly different from placebo group.	Resveratrol reduced CSF levels of TREM2, MMP-9, reduced markers of neuronal damage (e.g. NSE, PNF), reduced cathepsin D, altered angiogenin. Suggests anti-inflammatory and neuroprotective effect in AD
[55] Zhu et al., 2018.	RES (10 mg/day) + glucose (5 g) + malate (5 g) for 12 months.	To evaluate the effects of RES in MCI patients.	60 MCI patients.	≥50 years.	MMSE 18–26, ADAS-Cog 12–30.	MMSE, ADAS-Cog, NPI, CSF biomarkers.	No significant adverse effects reported.	No significant changes in MMSE, ADAS-Cog, or NPI scores compared to placebo. Results indicate no cognitive improvement with RES treatment.
[52] Turner et al., 2015.	RES (500–2000 mg/day) for 52 weeks.	To evaluate the effect of RES on cognitive function and Aβ levels in AD patients.	75 AD patients.	≥50 years.	MMSE < 24, ADAS-Cog ≥ 14.	MMSE, ADAS-Cog, Aβ40, Aβ42, tau, brain volume.	Nausea, diarrhea (like placebo group).	RES did not improve cognitive scores. (MMSE, ADAS-Cog), and Aβ levels were lower in the placebo group. It slowed brain volume loss, but no greater cognitive or functional benefit.
Saffron								
[79] Tsolaki et al., 2020.	Saffron (30 mg/day).	Cognitive impairment management in MCI.	<35 MCI patients.	50–85 years.	MMSE.	MMSE.	No adverse side effects.	Saffron improved the MMSE score in MCI patients compared to placebo.
[78] Farokhnia et al., 2014.	Saffron extract (30 mg/day).	Evaluating efficacy in AD treatment.	30 AD patients.	50–85 years.	SCIRS, FAST.	SCIRS, FAST.	No adverse effects.	Saffron had similar efficacy to memantine in reducing cognitive decline in AD.
Sesame								
[69] Jung et al., 2021.	Sesame oil cake extract (SOCE).	Cognitive improvement in MCI.	45 MCI patients.	50–85 years.	CNT, Aβ (1–40) levels.	CNT, Aβ levels.	No significant adverse effects.	SOCE improved cognitive function and reduced Aβ levels in MCI patients.
[84] Ito et al., 2018.	Sesamin (10 mg/day) and Astaxanthin (6 mg/day).	Cognitive function improvement in MCI.	50 MCI patients.	50–85 years.	Processing speed, task complexity.	Processing speed, task complexity.	Dizziness, cold, diarrhea.	The combination of sesamin and astaxanthin improved cognitive function in MCI patients.
Ginseng and *Ginkgo biloba*								
[45] Heo et al., 2012	Ginseng (SG-135, 4.5 mg/day).	Evaluate cognitive effects in AD patients.	40 AD patients.	65–85 years.	MMSE, ADAS-Cog.	MMSE, ADAS-Cog at 12 and 24 weeks.	Mild GI issues in 21% of participants.	SG-135 improved MMSE, ADAS-Cog, showing positive cognitive effects in AD.
[85] Kim et al., 2013	*Ginkgo biloba* (standardized extract).	Investigate neuroprotective effects in AD.	50 AD patients.	65–85 years.	ADAS-Cog, MMSE.	ADAS-Cog, MMSE, CDR-SB.	GI discomfort (minor).	*Ginkgo biloba* improved cognitive function, reduced amyloid deposition, neuroprotective.
[46] Yakoot et al., 2013	*Ginkgo biloba* (standardized extract).	Examine neuroprotective effects in AD.	30 AD patients.	60–80 years.	MMSE, ADAS-Cog.	MMSE, ADAS-Cog, CDR-SB.	Headache (minor), GI discomfort.	*Ginkgo biloba* enhanced brain circulation, improving cognitive function.
[86] Herrschaft et al., 2012	*Ginkgo biloba* extract EGb 761^®^ (240 mg/day).	Evaluate the efficacy and safety of EGb 761^®^ in dementia with neuropsychiatric features.	410 patients with dementia (Alzheimer’s or vascular).	≥50 years.	MMSE 10–24.	Neuropsychiatric Inventory (NPI), ADAS-Cog, CIBIC-Plus.	No serious adverse effects reported.	EGb 761^®^ significantly improved neuropsychiatric symptoms and cognitive function compared to placebo.
[87] Ihl et al., 2012	*Ginkgo biloba* extract EGb 761^®^ (240 mg/day).	Evaluate the efficacy and tolerability of EGb 761^®^ in AD and vascular dementia.	404 patients with Alzheimer’s or vascular dementia.	≥50 years.	MMSE 10–26.	ADAS-Cog, SKT, CGI.	No serious adverse effects reported.	EGb 761^®^ improved cognition and daily activities compared to placebo.
[88] Vellas et al., 2012	*Ginkgo biloba* extract EGb 761^®^ (240 mg/day).	Assess long-term use of EGb 761^®^ for preventing AD in elderly individuals with memory complaints.	2854 subjects without dementia.	≥70 years.	MMSE ≥26.	Incidence of AD, ADAS-Cog, CDR.	No significant differences in adverse events between groups.	No significant reduction in Alzheimer’s incidence with EGb 761^®^ compared to placebo.
*Curcumin*								
[91] Ringman et al., 2012	*Curcumin* (C3 Complex^®^ 2–4 g/day).	Assess *Curcumin*’s effect on cognitive decline.	60 AD patients.	65–85 years.	MMSE, ADAS-Cog, NPI.	MMSE, ADAS-Cog, NPI, CSF Aβ, tau, p-tau.	Minor GI issues (21%).	*Curcumin* did not significantly improve cognitive scores or Aβ biomarkers in AD.
*Melissa officinalis*								
[60] Noguchi-Shinohara et al., 2020.	*Melissa officinalis* (500 mg rosmarinic acid).	Investigating cognitive effects in mild AD.	23 AD patients.	60–80 years.	MMSE, ADAS-Cog, DAD, CDR.	MMSE, ADAS-Cog, NPI-Q.	No serious adverse events.	No significant cognitive improvement; NPI-Q improved irritability.
[93] Noguchi-Shinohara et al., 2023.	*Melissa officinalis* (500 mg rosmarinic acid).	Investigating effect on cognitive decline.	323 older adults.	65–85 years.	Clinical Dementia Rating (CDR).	CDR, cognitive function tests.	No serious adverse events.	*M. officinalis* reduced cognitive decline in adults without hypertension.
*Spirulina*								
[96] Choi et al., 2022.	*Spirulina maxima* (70% ethanol extract).	Investigate cognitive and memory improvement.	80 MCI patients.	50–80 years.	CNT, MoCA, Aβ biomarkers.	CNT, MoCA, plasma antioxidant capacity.	No significant side effects.	*Spirulina* improved visual, verbal memory, and antioxidant levels in MCI patients.
[97] Tamtaji et al., 2023.	*Spirulina* (500 mg/day).	Assess cognitive and metabolic effects in AD.	60 AD patients.	65–85 years.	MMSE.	MMSE, metabolic markers.	No significant side effects.	*Spirulina* significantly improved MMSE and metabolic parameters in AD patients.
*Citrus*								
[99] Galluzzi et al., 2022	*Citrus peel extract (rich in AUR and NAR).*	Investigate cognitive and biomarker effects.	50 older adults.	60–80 years.	Subjective cognitive decline (SCD).	Cognitive tests, biomarkers of oxidative stress.	No significant adverse events.	*Citrus* extract improved cognition and biomarkers in SCD patients.

AD: Alzheimer’s disease, aMCI: amnestic mild cognitive impairment, MCI: mild cognitive impairment, Aβ: amyloid β MMSE: Modified Mini-Mental State Examination, NPI-Q: Neuropsychiatric Inventory Brief Questionnaire Form, DSS: Digital Symbol Substitution (subtest of the Wechsler Adult Intelligence Scale), FAQ: Functional Activity Questionnaire, GDS: Geriatric Depression Scale, MoCA: Montreal Cognitive Assessment score, CM-SS: ADAS-Cognitive Chinese Medicine Symptom Scale, ADL: Activities of Daily Living, ADAS-Cog: Alzheimer’s Disease Rating Cognitive Scale, Ach: acetylcholine, AchE: acetylcholinesterase, Aβ42: amyloid-β protein 42, Tau: microtubule-associated protein tau, CDR: Clinical Dementia Rating, CDR-SOB: Clinical Dementia Rating Scale Sum of Boxes, HAMD: Hamilton Depression Rating Scale, AVLT: Auditory Verbal Learning Test, ROCF: Rey Osterrieth Complex Figure test, SNSB: Seoul Neuropsychological Screening Battery, CSF: cerebrospinal fluid, MRI: magnetic resonance imaging, MMPs: metalloproteinases, DSM-V: Diagnostic and Statistical Manual of Mental Disorders, FIM: Functional Independence Measure, PET: positron emission tomography, sVOI: standardized volumes of interest, SCIRS: Severe Cognitive Impairment Rating Scale; FAST: Functional Assessment Staging, FRSSD: Functional Rating Scale of Symptoms of Dementia, CNT: computerized neurocognitive function test, 8-OHdG: 8-hydroxy-2′-deoxyguanosine, CNSVS: Central Nervous System Vital Signs, DAD: Disability Assessment for Dementia scale, APMC: aloe polymannose multinutrient complex, SM70EE: Spirulina maxima 70% ethanol extract. RES: Resveratrol; CNT: Cognitive Neuroscience Test.

### 3.12. Overall Quality Assessment

The quality of the trials included was assessed using the Revised Cochrane risk-of-bias tool for randomized trials (RoB 2) [14], a summary of these assessments is presented in Table 5. 16 articles were rated as having a medium overall risk of bias [51,52,55,56,61,67,70,71,74,75,78,84,85,86,93], 11 as low overall risk [50,53,58,62,79,87,88,91,96,97,99], and 4 as high overall risk of bias [46,47,60,69]. Regarding bias arising from the randomization process, 31 studies were considered low risk. These studies clearly reported the methods used for generating the allocation sequence, assigned the randomization task to team members independent of data collection or outcome assessment, and concealed the allocation sequence until group assignment. Most of these were randomized controlled trials with double blinding, helping to minimize selection bias and ensuring assignment integrity [45,46,47,50,51,52,53,55,58,61,62,67,69,74,75,79,84,85,86,87,88,93,96,97,99]. Four studies lacked specific details about the randomization process, but baseline characteristics did not indicate selection bias and were thus considered medium risk [56,60,70,71]. One study was rated high risk due to contradictions across different sections of the manuscript [78]. In terms of blinding of participants, clinicians, and researchers, 19 studies were rated low risk as they followed double-blind procedures [46,50,51,52,53,58,62,67,69,78,79,84,86,87,88,91,96,97,100]. Ten studies did not provide clear details about outcome assessment blinding beyond the title and were categorized as medium risk [55,56,60,61,67,70,71,74,75,93]. Two studies were rated high risk because they lacked any blinding [46,85]. For bias due to missing outcome data, most studies were considered low risk, as they reported nearly complete data for randomized participants and used analysis methods that accounted for missing data [50,52,53,55,56,58,62,67,75,78,79,84,85,88,91,93,96,97]. 12 studies were rated medium risk because they included only partial data (approximately 80–85%) or did not specify how missing data were addressed [47,51,60,61,69,70,71,74,86,87,99]. One study was rated high risk for missing data without explanation [67]. Additionally, Galluzzi et al., 2022 [100] was rated high risk due to the absence of any mention of missing data handling or participant attrition. Regarding bias in outcome measurement, 18 studies were assessed as low risk because they used appropriate and consistent outcome measurement methods across groups [46,50,51,52,53,56,58,62,69,79,84,87,88,91,93,96,97,99]. Twelve studies used appropriate methods but did not blind outcome assessors, although the lack of blinding was unlikely to have influenced results; these were considered medium risk [47,55,60,61,67,70,71,74,75,78,85]. For bias in the selection of reported results, most studies followed pre-specified analysis plans and were therefore rated as low risk [45,46,50,51,52,53,55,56,58,60,61,62,69,70,75,78,79,84,85,93,94,95,96,97,99]. Six studies were rated medium risk because they compared outcomes only between baseline and final measurements without making between-group comparisons [47,67,74,86,87,88].

In conclusion, the evaluation of the included trials using the RoB 2 tool revealed that most studies were of medium risk of bias, mainly due to limitations in reporting of randomization, blinding, or management of missing data. A considerable number of trials were categorized as low risk, showing strong methodological rigor, including appropriate randomization, blinding, and data handling. However, a small subset of studies exhibited high risk of bias, largely due to methodological flaws, contradictory reporting, or lack of blinding. Overall, the methodological quality of the trials was generally adequate, with many employing rigorous design features that minimized bias and enhanced the trustworthiness of their findings. Nevertheless, the presence of studies with moderate to high risk of bias underscores the ongoing need for improved transparency and adherence to methodological standards in clinical research. Strengthening these aspects can improve the quality and reliability of evidence that informs clinical practice.

### 3.13. Molecular Mechanisms in Alzheimer’s Disease and Their Coverage in Clinical Trials

To provide a mechanistic framework for interpreting clinical interventions in AD, this section first summarizes the key molecular pathways involved in AD pathophysiology. Subsequently, we integrate both clinical trials identified in this review to these specific mechanisms, highlighting those that are already targeted, and preclinical evidence, highlighting how laboratory studies provide theoretical foundations for clinical observations, and where gaps remain in translational validation. This dual approach establishes a more comprehensive understanding of the molecular mechanisms of natural products in AD.

#### 3.13.1. Key Molecular Mechanisms in AD Pathology

AD is characterized by a complex interplay of several molecular processes [98,100]:

Cholinergic dysfunction: Reduction in acetylcholine due to increased acetylcholinesterase activity [101]. Preclinical studies have demonstrated that natural compounds such as *Huperzine A* act both as acetylcholinesterase inhibitors and NMDA receptor antagonists, thereby preserving cholinergic signaling and preventing excitotoxicity [102].

Oxidative stress and neuroinflammation are induced by Aβ aggregation and tau pathology [103]. *Curcumin* has consistently demonstrated antioxidant and anti-inflammatory effects in preclinical models, mitigating neuronal damage, reducing tau phosphorylation, and improving synaptic function [104,105]. Clinical trials with *Curcumin* (e.g., NCT99710, NCT164749) further confirm its potential, though bioavailability remains a limiting factor. *Citrus extracts* have also been shown to lower pro-inflammatory markers such as interleukin-8 (IL-8), aligning preclinical anti-inflammatory evidence with early clinical observations.

Aβ aggregation and tau hyperphosphorylation lead to senile plaques and neurofibrillary tangles [106]. Preclinical research highlights that plant-derived monomers can enhance autophagy-mediated Aβ clearance and reduce tau pathology [107]. Compounds such as *geniposide*, acting on the GLP-1 receptor, have reduced Aβ deposition and inhibited tau phosphorylation in animal models [108]. Clinical trials directly targeting these mechanisms with natural products remain limited, representing a key translational gap.

BBB disruption facilitates neurotoxicity and systemic inflammation [109]. Studies in animal models suggest that *olive oil polyphenols* preserve BBB integrity, reducing neuroinflammation and maintaining neuronal homeostasis [110]. Dynamic contrast-enhanced MRI has confirmed these effects in early human interventions, indicating a protective role of olive oil against neurovascular dysfunction in AD (e.g., NCT2921672 and NCT3824197).

Mitochondrial dysfunction and impaired autophagy contribute to reduced clearance of toxic proteins [111]. Preclinical work has identified natural peptides such as *humanin* that improve mitochondrial function and promote neuronal survival [102]. Evidence also supports the role of *resveratrol* in activating sirtuins, mimicking caloric restriction and protecting against protein aggregation. Clinical studies (e.g., NCT00678431) with resveratrol have validated its effects on aging-related pathways, though definitive outcomes in AD remain under investigation.

Calcium dysregulation and excitotoxicity are mediated by glutamatergic/NMDA signaling [112]. Preclinical models show that *Huperzine A* regulates calcium homeostasis by antagonizing NMDA receptors, while simultaneously enhancing cholinergic function [102,113]. These findings provide a mechanistic explanation for the cognitive improvements observed in small-scale clinical studies.

Insulin resistance in the brain alters glucose metabolism and exacerbates tau pathology [114]. Preclinical experiments with *geniposide* demonstrate modulation of insulin signaling through GLP-1R activation, reducing amyloid burden and tau phosphorylation [115,116]. This mechanistic evidence supports the rationale for clinical testing of GLP-1–targeting natural products in AD.

Reduced hippocampal neurogenesis limits cognitive recovery and plasticity [117]. Preclinical studies with *humanin* suggest it can promote neuronal survival and enhance neurogenesis in AD models, highlighting its potential as a therapeutic candidate [102]. Clinical translation of this mechanism is still lacking, underlining a significant research opportunity.

Neurotransmitter imbalance occurs especially in glutamatergic and dopaminergic pathways [118]. Animal studies with *Huperzine A* have demonstrated regulation of neurotransmitter release, contributing to synaptic stability and cognitive benefits [119]. These findings provide a mechanistic rationale for its use in clinical settings, where improvements in memory and cognition have been observed [119].

#### 3.13.2. Coverage of Mechanisms by the Reviewed Clinical Trials

**Figure 5:**
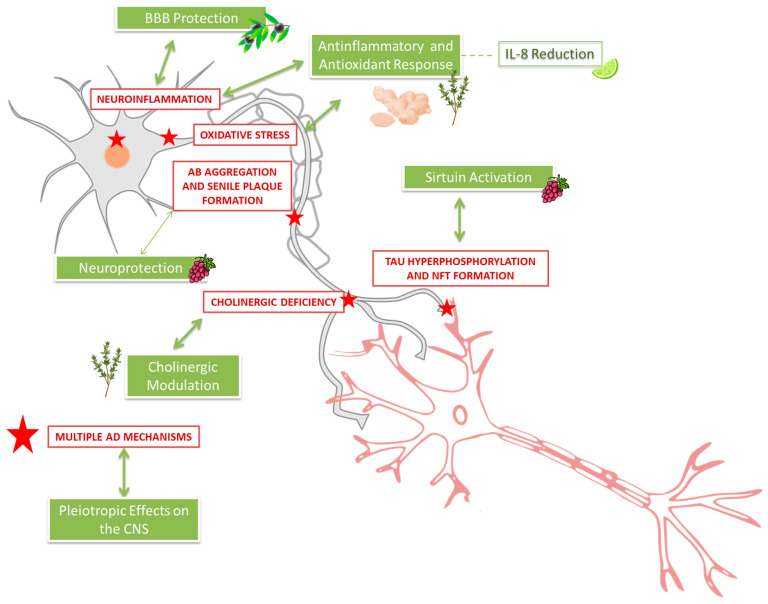
The relationship between AD p athological mechanisms and the therapeutic interventions targeting them. It visually connects key processes like amyloid-β aggregation, tau hyperphosphorylation, oxidative stress, neuroinflammation, and cholinergic deficiency to specific molecular mechanisms from clinical trials, such as cholinergic modulation (sage), antioxidant effects (*Curcumin* and sage), sirtuin activation (resveratrol), BBB protection (olive oil- and olive-tree-derived compounds ) and neuroprotection (grape-derived polyphenols). Aβ: amyloid β peptide; BBB: blood–brain barrier, CNS: Central Nervous System, IL-8: interleukin-8, NFT: neurofibrillary tangle. Green arrows indicate the benefit from the natural product and the pathway they regulate, while red stars indicate the problem or molecular mechanism characterized in Alzheimer’s disease.

The compiled data from clinical trials highlights the diverse molecular mechanisms targeted by various therapeutic interventions aimed at improving cognitive function and managing AD. Table 6 summarizes these mechanisms based on the clinical trials included in this systematic review (as detailed in Table 1), with a specific focus on molecular targets. Figure 5 further illustrates the relationship between AD pathological mechanisms and the therapeutic interventions designed to address them. It visually maps key pathological processes—such as Aβ aggregation, tau hyperphosphorylation, oxidative stress, neuroinflammation, and cholinergic deficiency—to specific molecular mechanisms targeted in clinical trials. Examples include cholinergic modulation (e.g., sage), antioxidant effects (e.g., *Curcumin*), sirtuin activation (e.g., resveratrol), and neuroprotective actions (e.g., grape-derived polyphenols). Abbreviations: Aβ—amyloid β peptide; BBB—blood–brain barrier; CNS—central nervous system; IL-8—interleukin-8; NFT—neurofibrillary tangle.

#### 3.13.3. Underexplored or Uncovered Mechanisms in Clinical Trials

Despite extensive preclinical evidence, several critical pathways in AD remain largely unexplored in human clinical trials involving natural compounds:

- Autophagy and proteasome function: Limited clinical data, though plant-derived monomers may enhance autophagy in preclinical models [120,121].
- Calcium homeostasis: Huperzine A shows promise but lacks comprehensive clinical validation [122,123].
- Insulin signaling dysfunction: Promising results from geniposide in preclinical models via GLP-1R activation [124,125].
- Hippocampal neurogenesis: Humanin and related peptides under investigation, no trials included here [116].
- Neurotransmitter modulation (non-cholinergic): Glutamate and dopamine regulation remain an open field [110,118].

### 3.14. Synthesis of Results

The synthesis included 31 clinical trials involving 3582 participants aged 50–90 years, with a mean treatment duration of 12.5 months. The review found that several natural compounds, particularly flavonoids, polyphenols, and omega-3 fatty acids, showed cognitive benefits in individuals with AD or MCI. Improvements were associated with reduced oxidative stress, inflammation, and Aβ levels. Some compounds (e.g., *Aloe vera*, *Spirulina*, citrus phytochemicals) showed strong cognitive and biomarker effects, while others (e.g., *Curcumin*, *Melissa officinalis*) had limited efficacy. Despite promising findings, heterogeneity in interventions, dosing, study design, and disease severity criteria limited the ability to perform direct comparisons. Overall, the results support the need for further investigation of these compounds in larger, standardized trials.

## 4. Conclusions

The 31 clinical trials-related articles included in the present systematic review involved 3582 patients (or participants), aged from 50 to 90 years. The mean duration of treatment was 12.5 months, with a minimum of 8 weeks’ and a maximum of 2 years’ follow-up. Many of the studies revealed favorable effects of natural compounds supplementation in improving the cognitive functions of AD or MCI patients; additionally, these natural compounds, such as flavonoids, polyphenols, and omega-3 fatty acids, are of particular interest due to their neuroprotective properties. The reviewed natural compounds showed promise in managing cognitive decline and AD through mechanisms such as reducing oxidative stress, inflammation, and Aβ levels. *Curcumin* and *Melissa officinalis* yielded limited cognitive benefits, while *Aloe vera*, *Spirulina*, and citrus phytochemicals demonstrated notable improvements in cognition and biomarkers. Ginseng and its combination with *Ginkgo biloba* showed potential in enhancing cognitive function, though more research is needed to validate these findings. Additionally, RES showed mixed results, with three of five studies reporting cognitive benefits but highlighting side effects such as weight loss and gastrointestinal issues. On the other hand, saffron demonstrated cognitive improvements, potentially through neuroprotective mechanisms, though findings are limited by small sample sizes. Finally, herbal supplements were often as effective as conventional treatments like donepezil, highlighting their therapeutic potential in AD and MCI. Nevertheless, inconsistencies in formulations and study designs highlight the need for large-scale, standardized trials to validate their effectiveness, determine optimal dosages, and better understand their mechanisms of action. In the same way, heterogeneity in terms of dosage, duration of the intervention, age of the participants, and degree of cognitive impairment makes it difficult to perform objective comparisons and draw concrete conclusions. In addition, some of the reviewed studies presented some limitations. Thus, criteria to establish the severity of the disease in participants were very heterogeneous and in some cases the degree of dementia was not declared. Many of the studies showed multiple biases that compromise their overall quality. In many of them, the small sample sizes per intervention group limited the statistical power. Likewise, most of the extracts were well tolerated, but in some studies the adverse effects found were not specified. Regarding molecular mechanisms, although current clinical trials with bioactive compounds address several key pathways involved in AD—such as cholinergic dysfunction, oxidative stress, inflammation, and BBB integrity—several critical mechanisms remain underexplored. Integrating preclinical findings, including those related to autophagy, insulin resistance, and neurogenesis, into clinical trial design is essential for developing multi-target, mechanism-based strategies for the prevention and treatment of AD. Future research should focus on investigating compounds that modulate these processes, designing rational combinations of bioactives with complementary mechanisms, and incorporating mechanism-specific biomarkers to validate molecular effects in vivo.

Given these challenges, there is a clear need for further large-scale, well-designed clinical trials to better understand the effects of natural compound supplementation on AD and MCI, addressing the limitations and biases observed in the current body of research. These should focus on standardizing protocols, ensuring larger and more diverse sample sizes, and providing clearer definitions of disease severity. While the current findings are promising, the translation of these results into clinical practice will require additional validation, including safety and efficacy data, as well as optimal dosing guidelines.

## 5. Limitations of the Study

This review is limited by the significant heterogeneity among the included clinical trials in terms of study design, participant age, cognitive impairment severity, treatment duration, and compound dosage, which hinders direct comparison and generalization of results. Many studies involved small sample sizes, reducing statistical power, and often lacked detailed reporting on adverse effects. Additionally, the criteria for diagnosing and classifying Alzheimer’s disease and MCI varied greatly, with some studies not clearly specifying dementia stages. While several natural compounds showed cognitive benefits, inconsistencies in formulation and reporting limit the ability to draw definitive conclusions. Furthermore, although key pathways such as oxidative stress and inflammation were addressed, other critical mechanisms like autophagy and neurogenesis remain underexplored. Future large-scale, standardized clinical trials with well-defined protocols and mechanism-specific biomarkers are necessary to validate these findings and determine optimal therapeutic strategies.

## Figures and Tables

**Table 1:** Criteria for selecting clinical trial articles.

Inclusion Criteria
- Completed clinical trials designed as randomized placebo-controlled study. - Studies targeting patients with AD or MCI. - Studies that correctly described the intervention design (groups, treatment duration, dose, evaluated outcomes). - Studies evaluating natural products. - Studies published in peer-reviewed journals.
Exclusion criteria
- Articles with incomplete data. - Studies on cognitively healthy subjects or with different pathologies other than AD or MCI. - Reviews, case reports, expert consensus, or papers with no full text available.

**Table 2:** Summary of completed clinical trials registered on ClinicalTrials.gov.

NCT	Conditions	Intervention Design	Outcomes	Study Population	Locations	Phase	Related Publications
Sage							
1001637	AD.	Sage or *Salvia officinalis* pills or no treatment during a 10-day period. Capsules taken by mouth.	-Tests to determine attention, memory, and visual cognition -EEG and ECG.	111 subjects (50–90 years old, all genders, no healthy volunteers).	Oregon, USA.	1	PMID: 12605619. PMID:2895683.
*Curcumin*							
00099710	AD.	Two different doses of *Curcumin* C3 Complex^®^ (2 g per day and 4 g per day) or a placebo, for the initial 6 months of the trial.	-Inflammation, oxidative damage, and cholesterol levels on blood and cerebrospinal fluid. -Cognition, behavior, and daily function.	33 subjects (>50 years old, all genders, no healthy volunteers).	California, USA.	2	PMID: 11571321. PMID: 23107780.
1716637	AD.	Etanercept (25 mg/week) for 6 weeks. Diet for 6/12 weeks: *Curcumin*, Omega-3, Quercetin, Resveratrol.	-Cognitive test.	12 subjects (60–85 years old, all genders, healthy volunteers).	Florida, USA.	1	PMID: 18644112.
164749	AD.	AD patients: placebo (1 g/day) or *Curcumin* (4 g/day), for six months. All: *Ginkgo biloba* leaf extract (120 mg/day).	-Cognitive test -Blood samples analyzed for levels of isoprostane, amyloid beta protein, metals, and cholesterol.	36 subjects (>50 years old, all genders, no healthy volunteers).	Hong Kong, China.	2	PMID: 18204357. PMID: 17951067.
*Ginkgo biloba*							
10803	AD. Dementia.	*Ginkgo biloba* (240 mg/day) for 6/12 weeks. Or EGb761^®^ (120 mg twice a day) for 6/12 weeks.	-MMSE. -ADAS-Cog. -Neuropsychological domains of memory, attention, visual-spatial construction, language, and executive functions, based on sums of z scores of individual tests.	>1500 subjects (>75 years old, all genders, healthy volunteers).	California, USA.	3	PMID: 20040554. PMID: 20123670. PMID: 19017911.
814346	AD.	EGb761^®^ (120 mg/day) for 18 months.	-18-Fluorodeoxyglucose-PET. -Cognitive tests—verbal fluency. -CDR. -GDS.	49 subjects (>65 years old, all genders, no healthy volunteers).	France.	2	Not provided.
42172	AD. Cognitive disorders.	Donepezil or placebo for the first 6 months. Donepezil + *Ginkgo biloba*.	-PET. -MRI.	40 subjects (>65 years old, all genders, healthy volunteers).	Iowa, USA.	4	Not provided.
1009476	AD. Dementia.	Galantamine (8 mg,16 mg, 24 mg) for 12 months. Nootropics in accordance with the recommendations.	-Tolerability. -Vital functions. -Global GDS. -MMSE. -Dementia-associated behavioral symptoms.	1134 subjects (>50 years old, all genders, no healthy volunteers).	Spain.	Not indicated.	Not provided.
Olive oil							
3824197	AD.	Extra-virgin olive oil enriched with oleocanthal, and other phenolic compounds added to daily diet. OR Olive oil with low phenolic content added to daily diet.	-Functional MRI imaging. -Blood–brain barrier function by dynamic contrast-enhanced MRI. -Cognitive tests.	25 subjects (55–75 years old, all genders, no healthy volunteers).	Alabama, USA.	Not applicable.	Not provided.
2921672	AD. Cognitive impairment.	Diet consisting of fruits, vegetables, grains, dairy, olive oils, seafood, and nuts for 9 weeks.	-Determine feasibility.	30 subjects (>65 years old, all genders, healthy volunteers).	Kansas, USA.	Not applicable.	Not provided.
Grape							
2502253	AD. Mild cognitive impairment.	Grape seed polyphenolic extract and resveratrol.	-Assessment of adverse events. -Levels of BDPP. -Neuropsychiatric Inventory and Cornell Scale for Depression in Dementia. -Memory, executive function, and attention measures.	14 subjects (50–90 years old, all genders, no healthy volunteers).	Maryland, USA.	1	Not provided.
1504854	AD.	500 mg RES by mouth once a day increasing at 13 weeks to a maximum of 1 g twice a day with or without food for one year.	-Volumetric MRI. -ADCS-ADL.	119 subjects (50–90 years old, all genders, no healthy volunteers).	26 different locations in USA.	2	PMID: 28086917. PMID: 33426901.
0678431	AD.	RES with glucose and malate. Dietary supplements are delivered in grape juice for one year.	-ADAS-Cog.	27 subjects (50–90 years old, all genders, no healthy volunteers).	New York, USA.	3	PMID: 30480082.
3361410	AD.	36 g of grape powder to be taken twice/day (total of 72 g/day) for 12 months or placebo.	-Regional cerebral metabolism, changes in neuropsychological performance measures.	>65 years old, all genders, no healthy volunteers).	LA, USA.	Not applicable.	Not provided.
Caffeine							
0726726	AD	5 mg Midazolam, 10 mg Warfarin, 10 mg vitamin K, 200 mg caffeine, 40 mg Omeprazole, 30 mg Dextromethorphan daily.	-Pharmacokinetic effects of BMS-708163 on interacting drugs. -Safety variables (adverse events, vital signs, safety labs).	22 subjects (18–45 years old, male, healthy volunteers).	New Jersey, USA.	1	Not provided.
0692510	AD.	Cocktail mix: CYP1A2 (caffeine), CYP2B6 (Bupropion), CYP2C8 (Rosiglitazone), CYP2C19 (Omeprazole), CYP3A4 (Midazolam), UGT1A1 (Bilirubin). Single dose of mix for 7 days.	-PK variables. -Safety variables (adverse events, vital signs, safety labs).	18 subjects (18–45 years old, male, healthy volunteers).	Sweden and UK.	1	Not provided.
4570085	AD.	Caffeine (100 mg/day) for 6 weeks, increasing after 3 weeks until 400 mg/day.	-Changes in NTB scores. -MMSE. -NTB subscores.	248 subjects (>50 years old, all genders, no healthy volunteers).	France.	3	Not provided.
Ginseng							
0391833	AD. Memory decline.	*Panax ginseng* powder (4.5 g/day) for 12 weeks.	-MMSE. -ADAS-Cog. -Biomarkers including hematopoietic progenitor cell count.	97 subjects (40 years old, female, no healthy volunteers).	Seoul, South Korea.	1 and 2.	PMID: 18580589.
03221894	AD.	One capsule/day in 150 mL warm water: 10 g ginseng, 30 g *Rehmannia glutinosa*, 10 g *Acorus tatarinowii*, 10 g *Polygala tenuifolia*, 10 g *Epimedium brevicornu*, 10 g *Cornus officinalis*, 10 g *Cistanche deserticola*, 10 g Curcuma aromatic, 10 g *Salvia miltiorrhiza*, 10 g Angelica sinensis, 10 g *Gastrodia elata*, and 10 g Berberine.	-MMSE. -ADCS-ADL. -CDR.	120 subjects (50–85 years old, all genders, no healthy volunteers).	Beijing, China.	Not applicable.	Not provided.
Citrus							
4744922	SCD.	One capsule extracts citrus a day for 9 months or placebo.	-Cognitive outcome (R-BANS). -Biological outcome (IL-8).	60 to 75 Years.	Italy.	Not aplicable.	PMID: 36253765. PMID: 25205962.

AD (Alzheimer’s disease), ADAS-Cog (Cognitive subscale of the AD Assessment Scale), ADCS-ADL (AD Cooperative Study-Activities of Daily Living), CDR (Clinical Dementia Rating), ECG (Electrocardiogram), EEG (Electroencephalogram), GDS (Geriatric Depression Scale), IL-8 (Interleukin 8), MMSE (Modified Mini-Mental State Examination), MRI (Magnetic Resonance Imaging), NCT (National Clinical Trial), NTB (Neuropsychological Test Battery), PET (Positron Emission Tomography), PMID (PubMed Identifier), R-BANS (Repeatable Battery for the Assessment of Neuropsychological Status), and SCD (Subjective Cognitive Decline).

**Table 3:** Cognitive tests summary table.

Cognitive Test	Key Domains/Aspects Evaluated	Normal Score Range/Interpretation
MMSE (Modified Mini-Mental State Exam).	Orientation, short-term memory, attention, visuospatial, language skills, understanding instructions.	≥25 = Normal; <24 = Cognitive impairment.
ADAS-Cog (Cognitive Subscale of ADAS).	Word recall, recognition, naming objects, following instructions, comprehension, ideational apraxia, attention, and orientation.	Higher scores (0–75) = Greater cognitive impairment.
NPI (Neuropsychiatric Inventory).	Neuropsychiatric symptoms (behavioral and psychological).	No standard score range, higher scores indicate greater severity of symptoms.
DSS (Digital Symbol Substitution).	Processing speed, cognitive flexibility.	No standard score range, lower scores indicate cognitive decline.
MoCA (Montreal Cognitive Assessment).	Attention, executive functions, memory, language, visuals, visuospatial abilities, orientation.	≥26 = Normal; <26 = Cognitive impairment.
GDS (Geriatric Depression Scale).	Depression symptoms in the elderly.	≥5 = Depressive symptoms likely.
ADAS-Cog Chinese Medicine Symptom Scale (CM-SS).	Cognitive function with a focus on a Traditional Chinese Medicine perspective.	No standard score range, higher scores indicate greater impairment.
CDR-SOB (Clinical Dementia Rating Scale Sum of Boxes).	Cognitive impairment, severity of dementia symptoms (e.g., memory, orientation, judgment).	Higher scores indicate more severe cognitive impairment.
AVLT (Auditory Verbal Learning Test).	Immediate and delayed word recall.	No standard score range, lower scores indicate greater memory impairment.
ROCF (Rey Osterrieth Complex Figure Test).	Visuospatial and memory functions.	No standard score range, lower scores indicate poorer visuospatial memory.
SNSB (Seoul Neuropsychological Screening Battery).	Various cognitive functions (attention, memory, language, visuals).	No standard score range, overall performance indicates cognitive ability.
FIM (Functional Independence Measure).	Functional ability (e.g., self-care, mobility, communication).	Higher scores indicate better functional independence.
SCIRS (Severe Cognitive Impairment Rating Scale).	Severity of cognitive impairment and related behaviors.	Higher scores indicate more severe impairment.
FAST (Functional Assessment Staging).	Stage of Alzheimer’s disease based on functional decline	Higher scores indicate later stages of AD.
CNSVS (Central Nervous System Vital Signs).	Cognitive functions (e.g., attention, memory, executive function).	No standard score range, lower scores indicate cognitive decline.

ADAS-Cog (Cognitive Subscale of the AD Assessment Scale), ADAS-Cog CM-SS (Cognitive Subscale of ADAS Chinese Medicine Symptom Scale), ADCS-ADL (AD Cooperative Study-Activities of Daily Living), AD (Alzheimer’s disease), AVLT (Auditory Verbal Learning Test), CDR-SOB (Clinical Dementia Rating Scale Sum of Boxes), CNSVS (Central Nervous System Vital Signs), DSS (Digital Symbol Substitution), ECG (Electrocardiogram), EEG (Electroencephalogram), FAST (Functional Assessment Staging), FIM (Functional Independence Measure), GDS (Geriatric Depression Scale), MMSE (Modified Mini-Mental State Examination), MoCA (Montreal Cognitive Assessment), MRI (Magnetic Resonance Imaging), NPI (Neuropsychiatric Inventory), NTB (Neuropsychological Test Battery), PET (Positron Emission Tomography), ROCF (Rey Osterrieth Complex Figure Test), SCIRS (Severe Cognitive Impairment Rating Scale), SNSB (Seoul Neuropsychological Screening Battery).

**Table 5:** Overall quality assessment of the studies included. Low risk of bias is represented in green, some concern in yellow, and high risk of bias in red.

Low risk of bias		Bias arising from the randomization process	Bias due to deviations from the intended interventions	Bias due to missing outcome data	Bias in measurement of the outcome	Bias in selection of the reported result	Overall risk of bias
Some concern							
High risk of bias							
Herbal extracts	[67]						
	[58]						
	[50]						
	[53]						
	[47]						
	[51]						
	[56]						
	[71]						
	[62]						
	[70]						
	[67]						
RES and grape	[74]						
	[75]						
	[61]						
	[55]						
	[52]						
Saffron	[79]						
	[78]						
Sesame related compounds	[69]						
	[84]						
Ginseng	[85]						
Ginseng + *Ginkgo biloba*	[46]						
*Ginkgo biloba*	[86]						
	[87]						
	[88]						
*Melissa officinalis*	[60]						
	[93]						
*Curcumin*	[91]						
*Spirulina*	[96]						
	[97]						
*Citrus*	[99]						

**Table 6:** Summary of molecular mechanisms in selected clinical trials.

Mechanism	Intervention(s)	Molecular Target	Clinical Trials
Cholinergic dysfunction	Sage extract	Cholinergic receptors (↑ ACh activity)	NCT1001637
Oxidative stress and inflammation	*Curcumin*, Resveratrol, Grape polyphenols, Citrus extract	ROS, IL-8, inflammatory cytokines	NCT0099710, NCT00678431, NCT4744922
BBB integrity	Extra virgin olive oil, Resveratrol	Cytokine-induced BBB disruption	NCT03824197, NCT1504854
Sirtuin pathway/aging	Resveratrol	SIRT1 activation, tau regulation	NCT1504854, NCT00678431
Amyloid and tau pathology	Grape polyphenols (indirect), possibly *Curcumin*	Aβ aggregation, tau phosphorylation	Indirect via biomarkers
Pleiotropic CNS effects	Caffeine, Citrus extract	Multiple neurotransmitter and cytokine pathways	NCT4570085, NCT4744922

ACh (acetylcholine), ROS (reactive oxygen species), IL-8 (interleukin-8), BBB (blood–brain barrier), SIRT1 (sirtuin 1), Aβ (amyloid-beta), and CNS (central nervous system). It also includes several NCT numbers, which refer to registered clinical trials in the ClinicalTrials.gov database. The symbol **↑** indicates an increase in ACh activity

## Data Availability

No new data were created or analyzed in this study. Data sharing is not applicable to this article.

## Supplementary Materials

The following supporting information can be downloaded at: https://www.mdpi.com/article/10.3390/ijms262110631/s1.

## Abbreviations

8-OHdG	8-hydroxy-2′-deoxyguanosine
Ach	Acetylcholine
AchE	Acetylcholinesterase
AD	Alzheimer’s disease
AD7C-NTP	Alzheimer-associated neuronal thread protein
ADAS-Cog	Cognitive subscale of the AD Assessment Scale
ADCS-ADL	AD Cooperative Study-Activities of Daily Living
ADL	Activities of Daily Living
aMCI	Amnestic mild cognitive impairment
APMC	Aloe polymannose multinutrient complex
AVLT	Auditory Verbal Learning Test
Aβ	Amyloid β-peptide
Aβ42	Amyloid-β protein 42
BHY	Bushenhuatanyizhi
BPSD	Behavioral and psychological symptoms of dementia
BSC	Bushen capsules
CDR	Clinical Dementia Rating
CDR-SOB	Clinical Dementia Rating Scale Sum of Boxes
CM-SS	ADAS-Cognitive Chinese Medicine Symptom Scale
CNSVS	Central Nervous System Vital Signs
CNT	Computerized neurocognitive function test
CRP	C-reactive protein
CSF	Cerebrospinal fluid
DAD	Disability Assessment for Dementia scale
DL	Davaie Loban
DSM-V	Diagnostic and Statistical Manual of Mental Disorders
DSS	Digital Symbol Substitution (subtest of the Wechsler Adult Intelligence Scale)
ECG	Electrocardiogram
EEG	Electroencephalogram
FAQ	Functional Activity Questionnaire
FAST	Functional Assessment Staging
FGF	Fibroblast growth factor
FIM	Functional Independence Measure
FRSSD	Functional Rating Scale of Symptoms of Dementia
GDS	Geriatric Depression Scale
GPx	Glutathione peroxidase
GSH	Glutathione
HAMD	Hamilton Depression Rating Scale
HYF	Huannao Yicong Formula
IL	Interleukin
JYF	Jiannao Yizhi Formula
KGT	Kami-guibi-tang
LDL	Low-density lipoprotein
MCI	Mild cognitive impairment
MDC	Macrophage-derived chemokine
MMP	Matrix metalloproteinase
MMPs	Metalloproteinases
MMSE	Modified Mini-Mental State Examination
MoCA	Montreal Cognitive Assessment score
MRI	Magnetic resonance imaging
NCT	National Clinical Trial
NFTs	Neurofibrillary tangles
NMDA	N-methyl-D-aspartate
NPI	Neuropsychiatric Inventory
NPI-Q	Neuropsychiatric Inventory Brief Questionnaire Form
NTB	Neuropsychological Test Battery
NYT	Ninjin’yoeito formula
PET	Positron emission tomography
PHF-tau	Paired helical filament
PON1	Paraoxonase 1
RES	Resveratrol
ROCF	Rey Osterrieth Complex Figure test
SCIRS	Severe Cognitive Impairment Rating Scale
SM70EE	*Spirulina maxima* 70% ethanol extract
SNSB	Seoul Neuropsychological Screening Battery
SOCE	Sesame oil cake extract
sVOI	Standardized volumes of interest
Tau	Microtubule-associated protein tau
TBARS	Thiobarbituric acid reactive substances
TNF-α	Tumor necrosis factor alpha
YHD	Yishen Huazhuo decoction
